# Cytotoxicity of Amphotericin B and AmBisome: *In Silico* and *In Vivo* Evaluation Employing the Chick Embryo Model

**DOI:** 10.3389/fphar.2022.860598

**Published:** 2022-06-08

**Authors:** Ahmad Khosravi, Iraj Sharifi, Hadi Tavakkoli, Elaheh Molaakbari, Sina Bahraminegad, Ehsan Salarkia, Fatemeh Seyedi, Alireza Keyhani, Zohreh Salari, Fatemeh Sharifi, Mehdi Bamorovat, Ali Afgar, Shahriar Dabiri

**Affiliations:** ^1^ Leishmaniasis Research Center, Kerman University of Medical Sciences, Kerman, Iran; ^2^ Department of Clinical Science, School of Veterinary Medicine, Shahid Bahonar University of Kerman, Kerman, Iran; ^3^ Department of Chemistry, Shahid Bahonar University of Kerman, Kerman, Iran; ^4^ Department of Anatomy, School of Medicine, Jiroft University of Medical, Sciences, Jiroft, Iran; ^5^ Obstetrics and Gynecology Center, Afzalipour School of Medicine, Kerman University of Medical Sciences, Kerman, Iran; ^6^ Research Center of Tropical and Infectious Diseases Kerman University of Medical Sciences, Kerman, Iran; ^7^ Research Center for Hydatid Disease in Iran, Kerman University of Medical Sciences, Kerman, Iran; ^8^ Afzalipour School of Medicine and Pathology and Stem Cells Research Center, Kerman University of Medical Sciences, Kerman, Iran

**Keywords:** leishmaniasis, amphotericin B, toxicity, apoptosis, angiogenesis, *in silico*, *in vivo*, chick embryo

## Abstract

Leishmaniasis has been identified as a significant disease in tropical and subtropical regions of the world, with Iran being one of the disease-endemic areas. Various treatments have been applied for this disease, and amphotericin B (Amp B) is the second line of treatment. Side effects of this drug have been reported in various organs. The present study investigated the effects of different types of Amp B on fetal organs using *in silico* and *in vivo* assays (chicken embryos). *In vivo* analysis was done by checking pathological changes, angiogenesis, and apoptosis alterations on eggs treated by Amp B and AmBisome. *In silico* approach was employed to predict the affinity of Amp B and AmBisome to the vascular endothelial growth factor A (VEGF-A), its receptor (KDR1), apoptotic-regulator proteins (Bcl-2-associated X protein (Bax), B-cell lymphoma (Bcl-2), and Caspase-8. The ADME-toxicity prediction reveals that AmBisome possesses a superior pharmacological effect to Amp B. The best result of all the dockings in the Molegro Virtual Docker (MVD) was obtained between Bax, Bcl-2, Caspase-8, KDR1, and VEGF-A targets. Due to the lower Egap (HOMO–LUMO) of AmBisome, the chemical reactivity of AmBisome was higher than that of Amp B. *In vivo* analysis showed that embryos that received Amp B exhibited less vascular density than AmBisome. Amp B alone significantly increased the expression of apoptosis and decreased angiogenesis genes compared to AmBisome. The histopathology analysis of the treated embryos showed a reduction in the blood vessel collapse and an increase in degenerative and apoptotic–necrotic changes in the embryonic tissues. Overall, the results suggest the potential benefits of AmBisome over Amp B, which might be a better treatment strategy to treat leishmaniasis during pregnancy.

## 1 Introduction

Leishmaniasis is a public disease in nearly 100 tropical and subtropical countries (Alvar et al., 2012). The causative agent of this disease is a protozoan parasite called *Leishmania*, which causes clinical manifestations in the form of cutaneous, mucocutaneous, and visceral (or kala-azar) (World Health Organization, 2020b). Cutaneous leishmaniasis is more common than other types and has been reported as an essential health issue in endemic regions, including Iran, Saudi Arabia, Syria, Afghanistan, Brazil, and Peru (World Health Organization, 2018). Based on the World Health Organization (WHO), about 12 million people are infected with this disease, and 1 million people are added to this number annually. Nearly 70 percent have been reported in 10 countries: Afghanistan, Iran, Syria, Sudan, Brazil, Peru, Costa Rica, Syria, and Saudi Arabia (World Health Organization, 2020a; Bahraminegad et al., 2021). Many determinants, including the human immunodeficiency virus (HIV) outbreak, a rise in international travel, the absence of efficacious vaccines, problems in controlling vectors, global conflicts, and the expansion of resistance to drugs, could upsurge the incidence of leishmaniasis (Sereno et al., 2007).

Nearly 25 chemical formulations display leishmanicidal activities in clinical practice; however, only a few have been confirmed worthy. The conventional therapy against leishmaniasis includes pentavalent antimonial derivatives (SbV). Other drug formulations, such as amphotericin B (Amp B), miltefosine, allopurinol, pentamidine, and aminosidine, can be used. The drugs used in leishmaniasis treatment present several problems, including high toxicity and many side effects, restricting patients from treatment, and the emergence of resistant variants (Singh and Sivakumar, 2004). Pentavalent antimonials have become the drug of choice to treat all types of leishmaniasis. Sodium stibogluconate (Pentostam^®^) was first used; meglumine antimonate (MA) can be used too (Brunton et al., 2018). Currently, several limitations have decreased antimonials: the inconstant efficacy against VL and CL and the increased antimonial resistance (Croft and Coombs, 2003). The recommendations have replaced the antimonials with Amp B in the treatment of leishmaniasis. In cases where resistant antimony strains emerge, the second-line drug is Amp B (Firooz et al., 2020).

Amp B is a macrolide polyene antifungal antibiotic agent; Amp B aims at the amastigote and promastigote membranes and is mainly given systemically. Contrary to miltefosine and pentavalent antimonials, amphotericin-B liposomal (AmBisome) is offered in many countries and permitted during gestation (Ramos et al., 1996; Morgan et al., 2007). Amp B can be directed intravenously and intralesionally and pervaded into surgical sites (Sidhu et al., 2018). Severe adverse effects have been reported by Amp B, including fever and chills, malaise, thrombophlebitis, and infrequent severe toxicities such as severe hypokalemia, myocarditis renal inadequacy, hypomagnesemia, metabolic academia, polyuria, and even death. Amp B deoxycholate has been reported for ventricular arrhythmias, bradycardia, and severe hypertension stated in overdoses with preexisting cardiac diseases, even when given in standard doses. Its use requires prolonged hospitalization and careful monitoring (Balaña-Fouce, 1998; Cavell, 2020). According to the FDA, Amp B is classified as a category B medication (Food and Drug Administration, 2011).

Various methods are used to evaluate the effects of a drug on fetal health. One of them is angiogenesis analysis, which plays a vital role in organ development, wound healing, fetal growth, placenta, and reproduction (Bamorovat et al., 2019a; Bamorovat et al., 2019b). It is necessary for cellular events such as migration, proliferation, differentiation of endothelial cells, and eventually vascular formation. Because of the importance of the angiogenesis process in many physiological and pathological processes, many researchers have studied angiogenesis in various laboratory models (Eskander and Tewari, 2014; Maroof et al., 2014). The chorioallantoic membrane of chicken embryos is a suitable model for evaluating the process of angiogenesis and its factors on fetus growth and development (Khosravi et al., 2019; Tavakkoli et al., 2019). The effects of medicine on the apoptotic process may also be used to evaluate the impact of a drug on embryonic health (Khosravi et al., 2018).

Given the therapeutic use of Amp B in the treatment of diseases such as leishmaniasis in various parts of the world and the fact that no study has been done on the effects of this drug on angiogenesis and apoptosis, the factors involved in apoptosis and angiogenesis under the influence of Amp B and AmBisome were investigated in this study using *in silico* molecular modeling and *in vivo* chick embryo assays.

## 2 Manuscript Formatting

### 2.1 *In Silico* Modelin*g*

#### 2.1.1 Visualization and Protein-Ligand Interaction Analysis

Predictive computational programs rely on a method known as macromolecular docking to identify the three-dimensional structures and intermolecular interactions between two molecules as they would usually appear in a biological system (Dominguez et al., 2003; Van Zundert et al., 2016). The protocols utilized to perform docking consist of two major components: a search algorithm and a scoring function (Damm-Ganamet et al., 2013). The search algorithm defines the three-dimensional shapes of the ligand and the protein as they are bound to each other (Kusumaningrum et al., 2014). The scoring function of the docking process specifies a numeric score to a specific three-dimensional protein-ligand structure that indicates the affinity between the two molecules. This scoring function is applied to rank the small molecules according to their ability to bind to the protein (Damm-Ganamet et al., 2013). The molecular docking approach was applied to use MVD software (Thomsen and Christensen, 2006). MVD is the most extensively utilized and practical tool in identifying ligand-protein exchanges and has higher correctness (87%) than other comparable tools. PDB records often have the underprivileged or lost assignment of obvious hydrogens, and the PDB file arrangement cannot offer bond order evidence. MVD automatically identifies probable binding spots (cavities or vigorous sites) by applying its cavity recognition algorithm. The cavities within a 30 × 30 × 30 Å^3^ cube positioned at the empirically recognized ligand position were utilized. The cavities detected by the cavity uncovering algorithm are then applied by the directed differential expansion search algorithm to emphasize the search to that special area undecided the docking replication (Kusumaningrum et al., 2014). The ultimate results were examined using Molegro Molecular Viewer 7.0, and the best interrelating complex was selected from each dataset.

Proteins involved in apoptotic-regulator proteins such as Bax, Bcl-2, and Caspase-8, as well as the regular expression of angiogenic-regulating genes such as KDR1 and VEGF-A, were used in this investigation.

**FIGURE 1:**
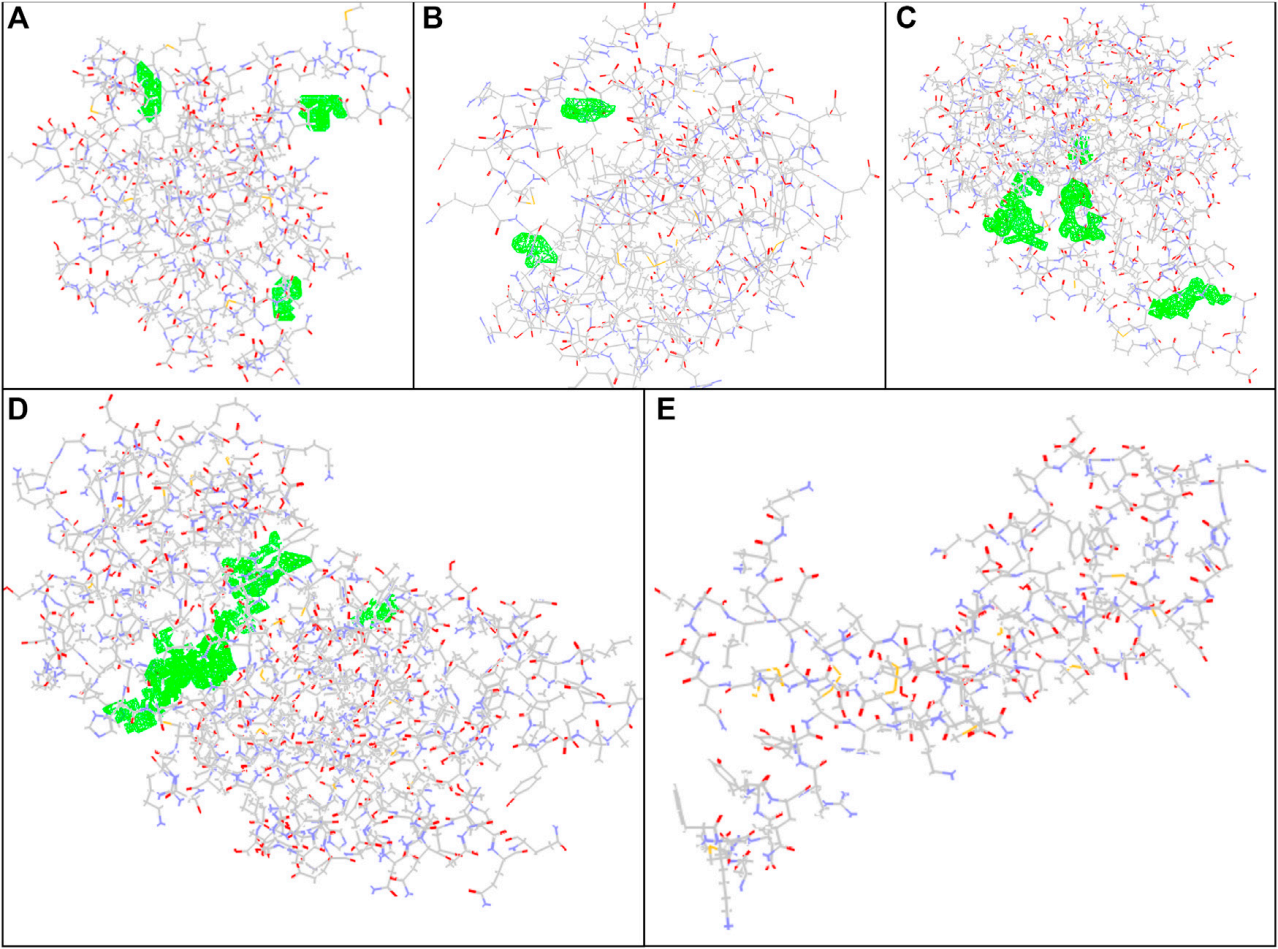
Docking conformation and active sites. **(A)** Bax, **(B)** Bcl-2, **(C)** Caspase-8, **(D)** KDR1, and **(E)**VEGF-A target proteins (PDB, ID, 5W5X, 5JSN, 1I4E, 2QU5, and 5t89, respectively) using MVD studies. Detected cavities; green; carbon atom; grey; oxygen atoms; red; nitrogen atoms; blue.

For preparing the molecular docking, supplementary structures in the PDB file were detached as various ligands in the downloaded file were removed using Discovery Studio software. In the case of the crystal structures for Bax, Bcl-2, Caspase-8, KDR1, and VEGF-A targets (PDB, ID, 5W5X, 5JSN, 1I4E, 2QU5, and 5t89, respectively), the package usually recognized distinct binding locations (Figure 1 **)**.

**FIGURE 2:**
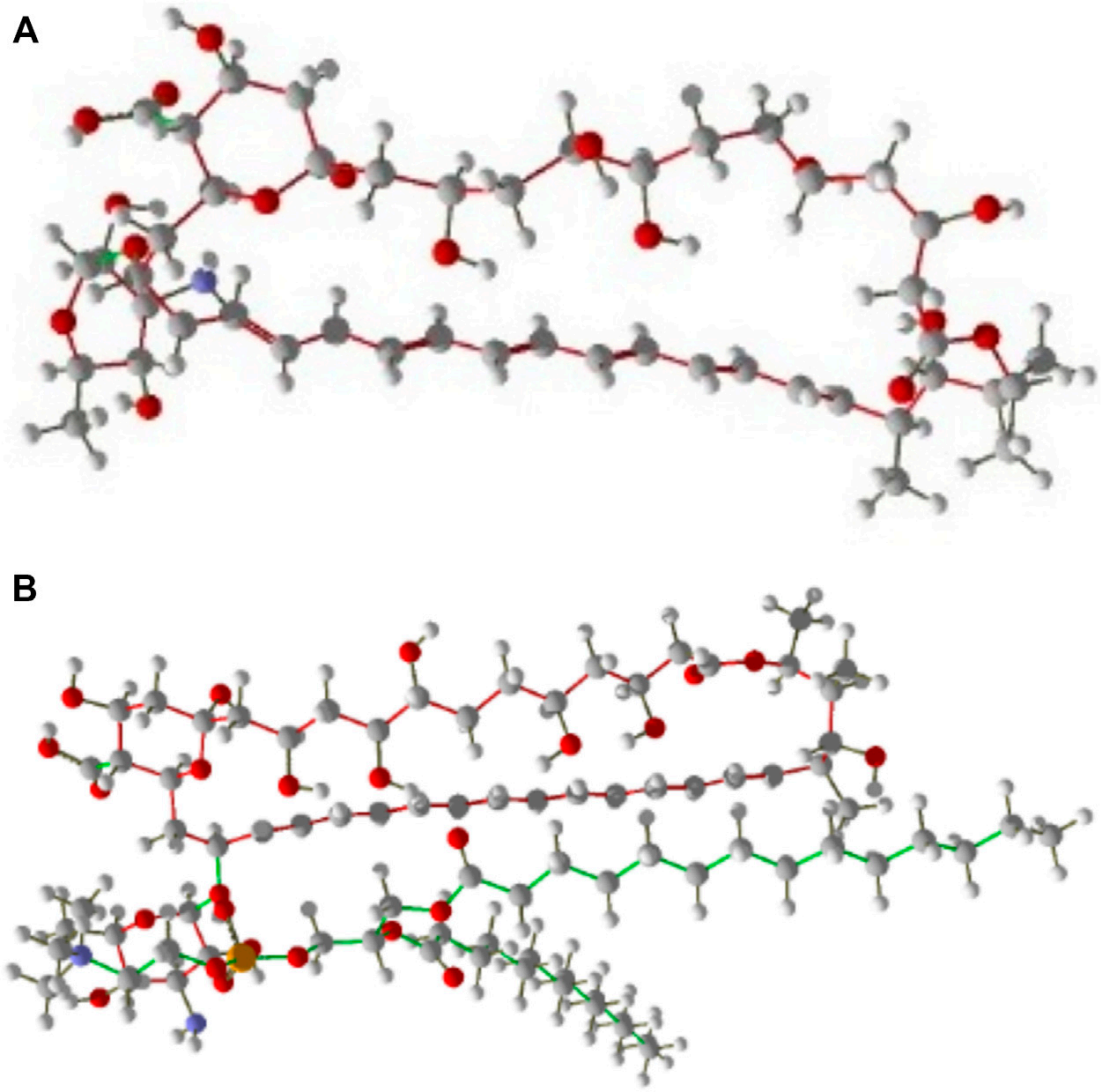
**(A)** Amp B, **(B)** AmBisome molecular structure as stick and bond type in position for MMV 7.0.

For designing different 3D structures of AmBisome, first, the conformers of the liposomes and Amp B (Figure 2A **)** were found in the National Center for Biotechnology Information (NCBI) PubChem compounds database (www.pubchem.ncbi.nlm.nih.gov/) and were transferred in SDF plan. The Materials Studio software was employed for molecular quantum designs through the DMol3 segment (Pelalak et al., 2021). Before the computational calculations, Hyperchem software was used to improve the geometry of Amp B and liposomes. Then, the DMol3 component was employed for geometry and energy optimization and the components’ HOMO and LUMO energy levels.

Three states were investigated to design and simulate the molecular structure of liposomes and Amp B:

1 Carbon chain region molecular structure of liposome with nitrogen ring and oxygenated Amp B.
2 The liposome’s hydrophilic region contains nitrogen and oxygen with the carbon chain of Amp B.
3 The hydrophilic heads and the hydrophobic portion of Amp B and AmBisome were placed side by side.

After that, optimization calculations and HOMO and LUMO calculations were performed. A combination of Amp B and AmBisome was chosen, and the possibility of their exchanges was assessed.

In every reproduction, a combination of two particles of Amp B and AmBisome were used to calculate the mixture’s low energy conformations and energy dispersal. Finally, the optimal structure with the least energy and the most stable Amp B and AmBisome was selected and saved in PDF format (Figure 2B).

#### 2.1.2 ADME and Toxicity Predictions

In drug discovery programs, computational programs were utilized to evaluate the ADME and harmfulness possessions at the primary stages of drug exposure. The highest engaged and the lowest vacant molecular orbitals (HOMOs and LUMOs, respectively), molecular surface area, hydrogen bond donor, hydrogen bond acceptor, and AlogP were calculated by Material Studio 15 software for AmBisome and Amp B. AdmetSAR is a free helpful source in the ADMET prediction of properties of new chemical substances such as for absorption, distribution, metabolism, and excretion studies (Yang et al., 2019).

### 2.2 *In Vivo* Examination

In this grouping, the anti-angiogenic possessions of Amp B were appraised in the chick embryo assay using yolk sac membrane (YSM) and molecular assays.

#### 2.2.1 Materials

According to Mahan Breeder Farm, Kerman, Iran, and fertile chicken eggs (Ross 308, regular weight of 55.8 ± 0.7 g). The breeder flock was reserved under ideal background circumstances. Amp B was obtained from Health Biotech Ltd. (Solan, India) and melted in sterile phosphate-buffered saline (PBS). A standard solution was provided and kept in small aliquot parts for upcoming use. PBS and paraffin were attained from Merck, Darmstadt, Germany. RNA extraction kit and SYBR Green Kits (FastStart SYBR Green Master) were bought from Roche Diagnostics GmbH, Mannheim, Germany. cDNA synthesis kit was purchased from BIONEER, Seoul, South Korea.

#### 2.2.2 Drug

Conventional and liposomal forms of amphotericin B were supplied from Health Biotech Ltd., India, and BDR Pharmaceuticals India, respectively. Each vial of both formulations contains 50 mg Amp B. The current study used different dosages of Amp B (1 and 2 mg/per kg of egg weight, which are the equal and double concentrations of therapeutic dosages employed during pregnancy, respectively) to investigate the impact of the drug on vascular apoptosis (Figueiro-Filho et al., 2004).

#### 2.2.3 YSM Assay

The YSM assay was carried out to assess the *in vivo* anti-angiogenic likelihood of Amp B. Fertile chicken eggs (Ross 308) were incubated at 37°C ± 1 in 75% humidity. Eggshells were cleansed with ethyl alcohol (70%), and a hole was completed over the rounded opposite of the shell. As a control, 50 µl of either Amp B or PBS was thrown down in the shell membrane. Amp B 1 was used at a concentration of 160 mg per kilogram. The fertilized eggs were re-injected two separate times after the first injection: 24 and 48 h later. The eggshells were sealed with molten paraffin, and the eggs were pre-incubated under the same conditions as before. The eggshell and shell membrane were aseptically separated to expose the surface of the YSM by breaking a 25 mm window on the egg on the Hamburger–Hamilton growth stage 22–24 (day 4 of the incubation period). Each group received a minimum of 10 eggs. A stereomicroscope (Luxeo 4D Stereozoom Microscope, Labomed, CA, United States) was used to capture high-quality images (4,000 × 3,000 pixels) that were analyzed on a 14.5-inch PC (Intel Core i3-390M, 2.66 GHz). The YSM was then cut (2.5 cm diameter) with scissors, and neighboring embryonal tissues were surgically removed. A molecular assay was performed on the sliced YSM.

#### 2.2.4 Molecular Assay

Quantitative real-time PCR (qPCR) reaction was performed to assess the effect of Amp B on angiogenesis and vascular apoptosis. The assay is described as follows: the YSM (*n* = 6) were first dissected from embryos, and relative expression levels of Caspase-8, Apaf1, AIF1, Bax, Bcl-2, VEGE-A, and KDR genes were determined by qPCR assay. According to the suggested protocol, the RNeasy^®^ small kit isolated total RNA from the extra-embryonic membrane (Qiagen, Chatsworth, CA). The NanoDrop ND-1000 was used to measure RNA concentration (ng) and purity (260:280 nm) spectrophotometrically (NanoDrop ND-1000, Thermo Scientific, Wilmington, DE, United States). The quantitative PCR (qPCR) method was developed using an SYBR Green assay (SYBR Premix Ex Taq TM II, Takara Bio, Inc, Shiga, Japan) with the Rotorgene Cycler system after reverse transcription at 37°C for 15 min (Corbett Research, Sydney, Australia).

The specific primers and reference gene sequences are listed in Table 1. After an initial step of 95°C for 1 min, 40 cycles of amplification were carried out. Each cycle consisted of 10 s at 95°C for DNA denaturation, 15 s at 60°C for primer annealing, and 20 s at 72°C for an extension. The expression levels of the selected reference gene were used to calculate the expression levels. The comparative Ct (2-Ct) approach was used to calculate expression levels.

**TABLE 1:** The specific primers and reference gene sequences for quantitative real-time RT-PCR.

Template	Forward primer	Reverse primer
Caspase-8	TCC​TCT​TGG​GCA​TGA​CTA​CC	TGT​CAA​TCT​TGC​TGC​TCA​CC
Apaf1	TTG​CCA​ACC​AGA​GAC​ATC​AGA​GG	TGC​GGA​CGA​ACA​ACC​AGA​CG
Bax	CCC​GAG​AGG​TCT​TTT​TCC​GAG	CCA​GCC​CAT​GAT​GGT​TCT​GAT
Bcl2	AGC​GTC​AAC​CGG​GAG​ATG​T	GCA​TCC​CAT​CCT​CCG​TTG​T
AIF1	GCG​TTA​ATG​TTT​ATA​TGC​CTA​ATG	CCTCCGAAGTCAGAATCC
VEGF	CAA​TTG​AGA​CCC​TGG​TGG​AC	TCT​CAT​CAG​AGG​CAC​ACA​GG
KDR1	GGA​GTT​TCC​CAG​AGA​CCG​AC	CAA​TCC​CAA​AGG​CAT​CAG​C
HPRT	GAT​GAA​CAA​GGT​TAC​GAC​CTG​GA	TAT​AGC​CAC​CCT​TGA​GTA​CAC​AGA​G
GAPDH	CCT​CTC​TGG​CAA​AGT​CCA​AG	GGTCACGCTGGAAGATA

#### 2.2.5 Histopathological Evaluation

The tissue samples were preserved in a 10% buffered formalin solution and embedded in paraffin. Five mm thick samples were sectioned by the microtome (Slee-Germany) on a thickness of 5 µm for histopathological examinations and stained with hematoxylin and eosin (H&E). The markers Bcl-2 (mouse monoclonal antibody, American, code number: PDMO16- lot No. H147) and Bax (Zytomed Germany, code number: 502-17990) were then stained immunohistochemically (IHC). The mean number of positively stained cells in 10 high-power fields was used to compute Bcl-2, Bax, and CD34 expression levels.

### 2.3 Statistical Analysis

SPSS software, version 20, was used for statistical analysis (SPSS Inc., Chicago, IL, United States). The statistical significance of changes in vascular limitations and gene expression levels was investigated using a one-way ANOVA test. A statistically significant *p*-value of 0.05 was used to analyze the data.

YSM analysis was performed by software packages including ImageJ^®^ 1.48 (National Institutes of Health, Bethesda, Maryland, United States) and MATLAB^®^ (Mathworks MATLAB R2015a). An area of interest (310 mm^2^ comprising 13203084 pixels) was discovered in the vitelline plexus after an area (28003000 pixels) was excised from the collected pictures. In the selected images, the vascular density was computed.

## 3 Results

### 3.1 *In Silico* Modeling

#### 3.1.1 Molecular Docking Studies

**FIGURE 3:**
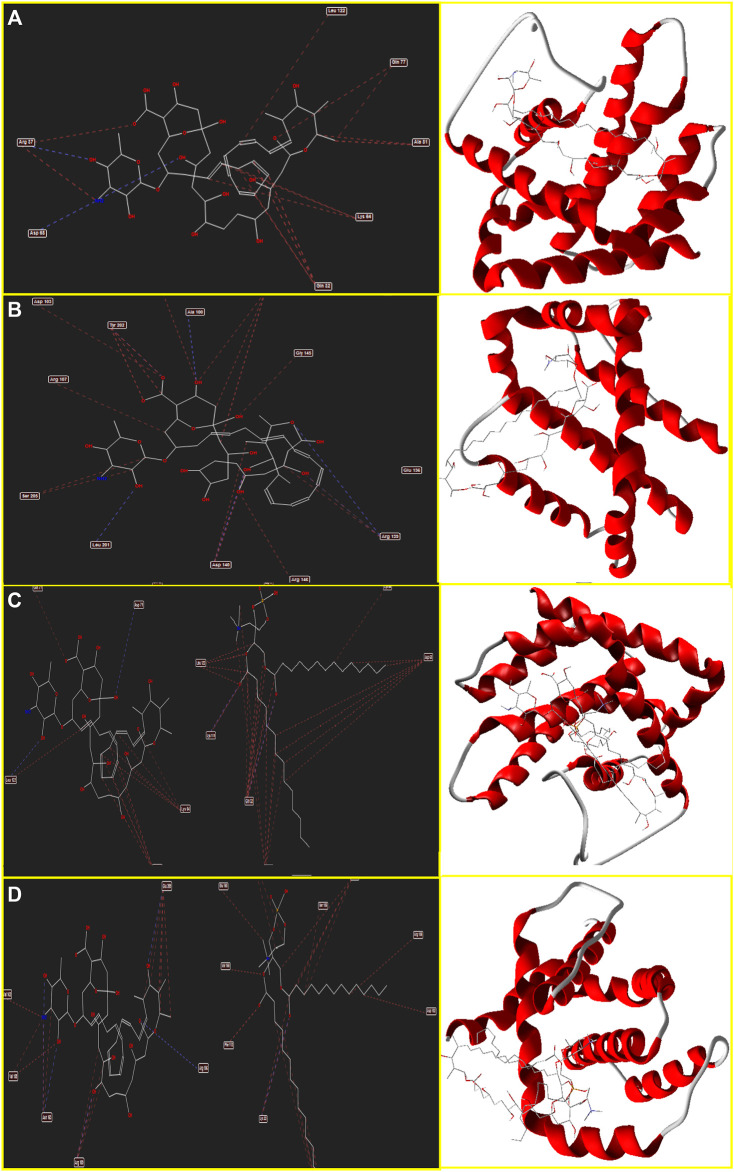
Schematic of the best score docking solution of the Amp B ligands and **(A)** BAX, **(B)** Bcl-2 target and AmBisome, **(C)** BAX and **(D)** Bcl-2 target with the selected crystal structure of 5W5X and 5JSN, respectively, along with the pharmacophore and ligand map of MMV 7.0.

The binding energy and interaction of amino acid residues are shown in Table 2. As shown in Figure 3, an Amp B displayed hydrogen bonding and steric interaction with Gln 32, Arg 37, Lys 64, Asp 68, Gln 77, Ala 81, and Leu 122 to Bax, with a Bax docking score of −141.37 kcal/mol. AmBisome binds to Bax (Figure 3C) with a docking score of −218.57 kcal/mol, and the binding site consists of amino acid residues such as Gln 32, Asp 63, Lys 64, Asp 71, Gln 77, Lys 119, and Leu 122 with H bond interaction and steric interaction. The resulting data from docking analysis in Figure 3B showed that Amp B forms H-bonds and steric interactions with amino acids of the Bcl-2 using Arg 106, Arg 109, Val 156, Val 159, Asp 163, Glu 160, and Glu 209 with docking −119.43 score kcal/mol. Also, AmBisome was stabilized by Bcl-2 (Figure 3D) with a docking score of −184.35 kcal/mol and using H bonds interaction and steric interactions with amino acid residues: Lys 22, Arg 26, Asp 102, Ser 105, Arg 106, Arg 109, Phe 112, Val 156, Val 159, Glu 160, Val 162, Asn 163, and Glu 209.

**TABLE 2:** Parameters from the interaction between the Amp B and AmBisome ligands and BAX, Bcl-2, Caspase-8, KDR1, and VEGF-A receptor of MVD.

Compound	Docking score for amphotericin B	Docking score for AmBisome
Bax	−141.37	−218.57
Bcl-2	−119.43	−184.35
Caspase-8	−178.08	−283.09
KDR1	−168.45	−313.05
VEGF-A	−155.37	−245.11

**FIGURE 4:**
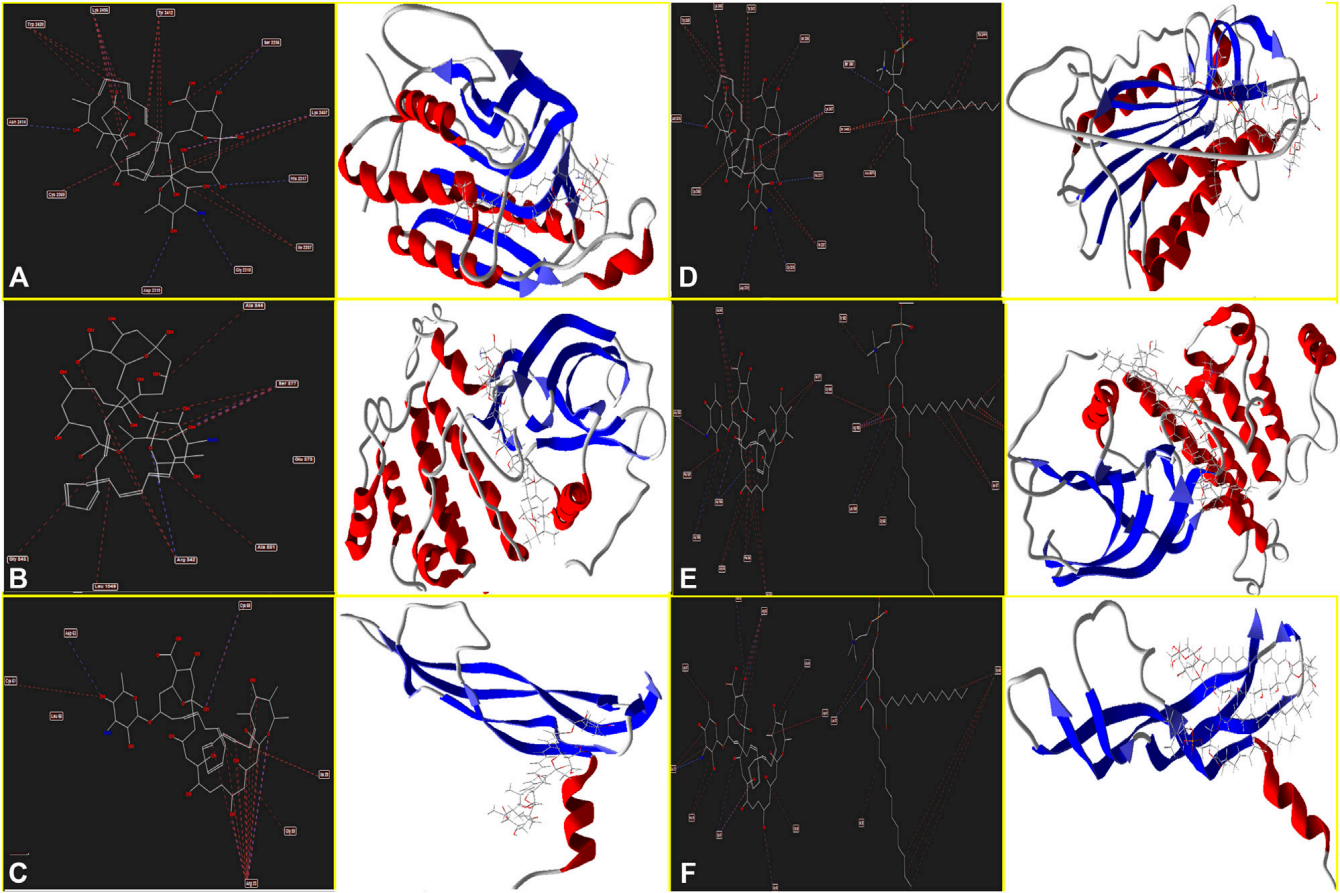
Schematic of the best score docking solution of the Amp B ligands and **(A)** Caspase-8, **(B)** KDR1, **(C)** VEGF-A target and AmBisome, **(D)** Caspase-8, **(E)** KDR1, and **(F)**VEGF-A target with the selected crystal structure of 1I4E, 2QU5, and 5t89, respectively, along with the pharmacophore and ligand map of MMV 7.0.

Moreover, Figure 4A determines Amp B forms H-bonds and steric interactions with amino acids of the Caspase-8 using Ser 2256, Ile 2257, Asp 2259, His 2317, Gly 2318, Asp 2319, Cys 2360, Tyr 2412, Asn 2414, Trp 2420, and Lys 2456 with a docking score of −178.08 kcal/mol. Consequently, AmBisome uses H bonds interaction and steric interactions with amino acid residues: Ile 2369, Pro 2370, Val 2371, Thr 2444, Asn 2447, Ser 2451, Asn 2452, Asp 2454, Lys 2461, and Met 2463 of Caspase-8 (Figure 4D) with a docking score of −283.09 kcal/mol.

Hydrogen bond interactions and steric interactions of Amp B and AmBisome with KDR1 and VEGF-A are shown in Figures 4C–F. Furthermore, Table 2 shows that the Docking scores of Amp B interacting with KDR1 and VEGF-A were −168.45 and −155.37, respectively. KDR1 and VEGF-A docked well into AmBisome, and their docking scores were −313.05 and −245.11 kcal/mol, respectively. Figure 4F shows Amp B docked with KDR1, and their residues were Arg 842, Gly 843, Ala 844, Ser 877, Ala 881, and Leu 1049. Also, AmBisome binds to KDR1 with a binding site consisting of amino acid residues such as Glu 818, Arg 842, Gly 843, Ala 844, Phe 846, Ser 877, Arg 1032, Phe 1047, Gly 1048, Leu 1049, Arg 1066, Tyr 1082, Asp 1084, and Arg 1088 with H bonds interaction and steric interaction (Figure 4E). The residues Arg 23, Ile 29, Gly 59, Cys 60, Cys 61, and Asp 63 of VEGF-A interact with Amp B by H bond and steric interactions. AmBisome binds to the active site of VEGF-A with a binding site consisting of amino acid residues such as Glu 38, Arg 56, Cys 57, Gly 59, Cys 60, Glu 64, Pro 70, Glu 73, and Leu 97 with H-bonds and steric interactions (Figure 4C). The docking results are shown in Figure 4F that AmBisome was more effective than Amp B. Pertinent tables, energy, and molecular docking scorings presented that AmBisome provides favorable docking results, as demonstrated by their considerable scoring functions and high protein-ligand interaction energy, which simultaneously indicated its affinity to the targets. As a consequence of the docking results, AmBisome illustrates more desirable antileishmanial activities than Amp B.

#### 3.1.2 Molecular Calculations

The results of HOMOs and LUMOs, molecular surface area, hydrogen bond donor, hydrogen bond acceptor, and AlogP were calculated by Material Studio 15 software for AmBisome and Amp B in Table 3. The equivalent energy gaps between HOMO and LUMO (Eg = ELUMO − EHOMO) were about 4.9593 and 7.0647 eV for AmBisome and Amp B molecules, respectively. The AlogP process has many uses, such as estimating local hydrophobicity, visualizing molecular hydrophobicity maps, and evaluating hydrophobic relations in protein-ligand multiplexes (Ghose et al., 1998). The superficial extent in A ° 2 indicates the ideal interaction of the acceptor atoms of a molecule with an H-bond donor probe and an H-bond acceptor. The H-bond acceptor capability and the H-bond donor ability play significant roles in absorption (Raevsky and Skvortsov, 2005). Due to the lower HOMO–LUMO energy gap, higher molecular surface area, hydrogen bond acceptor, and AlogP value of AmBisome compared to Amp B molecules, the reactivity can be concluded with the absorption of AmBisome molecule higher than that of Amp B molecule. Due to less HOMO–LUMO, Egap of AmBisome, the chemical reactivity was of AmBisome higher than that of Amp B.

**TABLE 3:** Molecular properties of the compounds.

Compound	HOMO (eV)	LUMO (eV)	Egap^a^ (eV)	Molecular surface area (A°)	Hydrogen bond donor	Hydrogen bond acceptor	AlogP
Amphotericin B	−7.87	−0.812	7.06	1.07	13	17	3.339
AmBisome	−6.88	−1.93	4.95	1.92E0	13	18	3.433

a Egap = ELUMO–EHOMOeV.

#### 3.1.3 ADMET Prediction

Table 4 shows the various ADMET parameters obtained using the admetSAR tool. ADMET characteristics determined that AmBisome has a greater Human Intestinal Absorption (HIA) score than Amp B, as inferred from the admetSAR server. AmBisome considers penetration of the blood–brain barrier (BBB) to be the best. When predicting the efflux by P-glycoprotein (P-gp), AmBisome is considered a substrate and inhibitor of P-gp, while Amp B is considered a substrate and non-inhibitor of P-gp. In metabolism terms, we found that AmBisome and Amp B were a non-substrate (but a non-inhibitor) of the CYP450 microsomal enzyme. A non-inhibitor of CYP450 indicates that the molecule will not prevent the biotransformation of drugs metabolized by the CYP450 enzyme. A test of AMES toxicity is applied to identify whether a compound is mutagenic or not. Both the AmBisome molecule and Amp B passed the AMES toxicity test, indicating that neither ligand is mutagenic.

**TABLE 4:** ADMET properties of the compounds.

Model	Compound			
Absorption	Amphotericin B		AmBisome	
Blood–brain barrier	BBB-	0.9659	BBB-	0.9799
Human Intestinal Absorption	HIA-	0.9308	HIA-	0.9996
Caco-2 permeability	Caco2-	0.7539	Caco2-	0.6622
P-glycoprotein Substrate	Substrate	0.6404	Substrate	0.8945
P-Glycoprotein Inhibitor	Non-inhibitor	0.7322	Inhibitor	0.6358
Distribution				
Subcellular localization	Mitochondria	0.4007	Lysosome	0.5372
Metabolism				
CYP450 2C9 Substrate	Non-substrate	0.7916	Non-substrate	0.7916
CYP450 2D6 Substrate	Non-substrate	0.8333	Non-substrate	0.8333
CYP450 3A4 Substrate	Substrate	0.6065	Substrate	0.6065
CYP450 1A2 Inhibitor	Non-inhibitor	0.8200	Non-inhibitor	0.8200
CYP450 2C9 Inhibitor	Non-inhibitor	0.7974	Non-inhibitor	0.7974
CYP450 2D6 Inhibitor	Non-inhibitor	0.8721	Non-inhibitor	0.8721
CYP450 2C19 Inhibitor	Non-inhibitor	0.7602	Non-inhibitor	0.7602
CYP450 3A4 Inhibitor	Non-inhibitor	0.7455	Non-inhibitor	0.7455
CYP Inhibitory Promiscuity	Low CYP Inhibitory Promiscuity	0.9878	Low CYP Inhibitory Promiscuity	0.9878
Toxicity				
	Non-inhibitor	0.7887	Non-inhibitor	0.5227
AMES toxicity	Non-AMES toxic	0.9133	Non-AMES toxic	0.6785
Carcinogens	Non-carcinogens	0.9682	Non-carcinogens	0.9002
Acute oral toxicity	III	0.7227	III	0.5889
Carcinogenicity (three-class)	Non-required	0.4873	Non-required	0.5044
ADMET predicted profile—regression				
Aqueous solubility	−3.0909	LogS	−3.0575	LogS
Rat acute toxicity	2.2357	LD50, mol/kg	2.7205	LD50, mol/kg

The ligands were also found to be noncarcinogenic in the carcinogenic profile. The MES toxicity test indicates that both ligands are non-mutagenic. The ligands were also found to be noncarcinogenic in the carcinogenic profile. Both ligand compounds have a low and comparable oral toxicity. Essential data from the admetSAR service were used to compute the LD50 dose in a rat model. A substance with a lower LD50 dose is more lethal than one with a higher LD50 value. We discovered that AmBisome had differing LD50 (2.72 vs. 2.23, respectively) compared to Amp B. Amp B had the lower LD50 and was more toxic than AmBisome. A log S value measures solubility; the lower the log S value, the higher the solubility, which improves absorption. As a result, AmBisomes with lower log S absorb more efficiently than Amp B. The ADMET evaluation is required to indicate the pharmacokinetic profile of the potential compounds, whereas the bioavailability of those in the body is determined *via* their physicochemical properties. The recognition of absorption, distribution, metabolism, excretion, and toxicity is involved in the identification of the pharmacokinetics of a drug. The data on ADME-Toxicity in Table 4 provided that AmBisom possesses the optimal pharmacological effect in comparison to Amp B. Nevertheless, Amp B includes low permeability and would be degraded in the stage of membrane absorption due to its hydrophilic properties that lead to a reduction in its bioavailability. As a result, encapsulation employing liposomes as the drug carrier can be utilized to improve the Amp B permeability and bioavailability.

### 3.2 *In Vivo*

#### 3.2.1 Effect of Amp B on the Vascular Density

**FIGURE 5:**
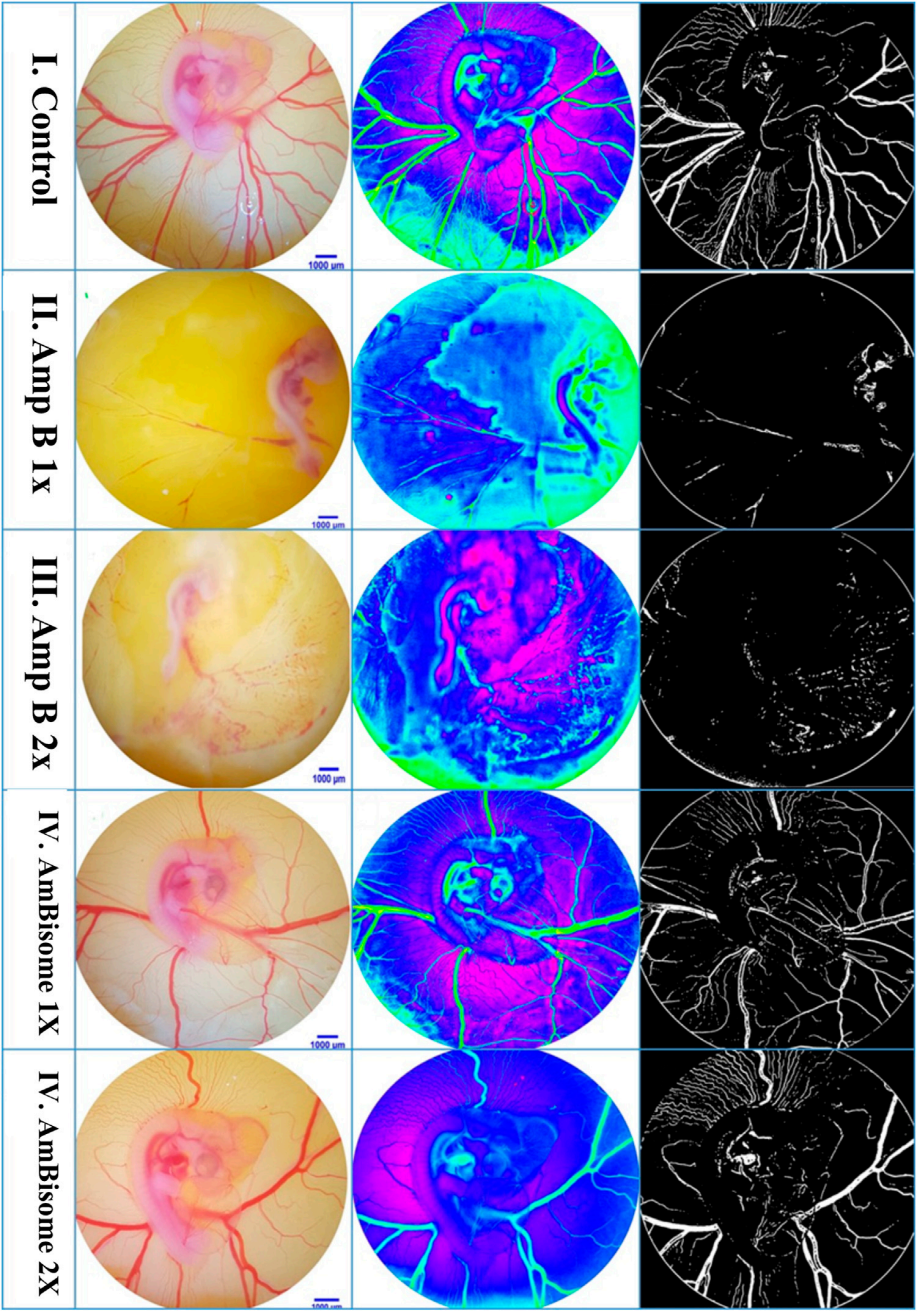
Amphotericin B and AmBisome affect the blood vessel system. (I) Control embryo with typical blood vessel system. (II and III) Amp B 1x and 2x, respectively. The blood vessel system is disrupted. (IV and V) AmBisome 1X and 2X, respectively. A smaller decrease in vascular density was exhibited compared to Amp B.

**FIGURE 6:**
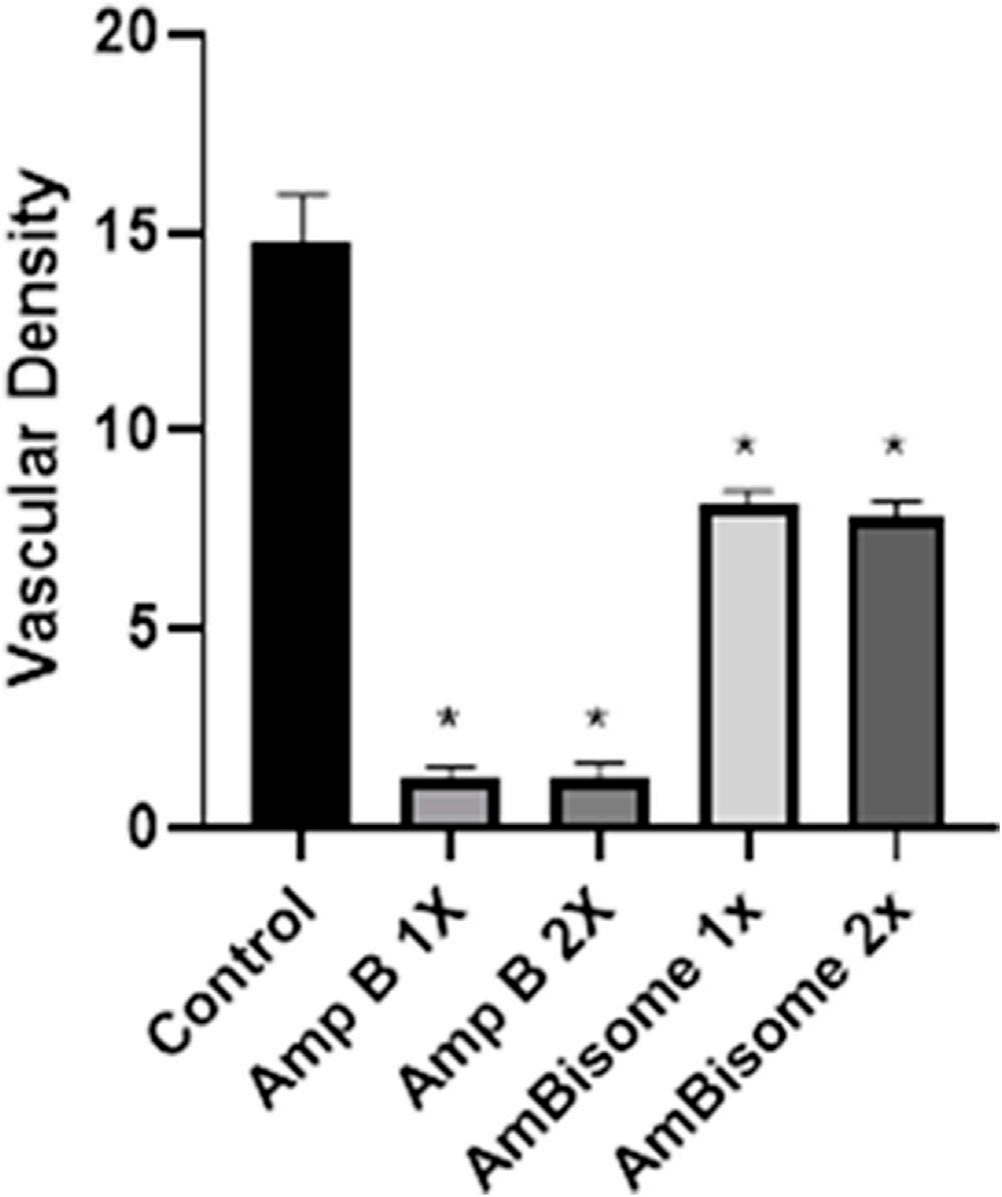
Vascular density following Amphotericin B and AmBisome. Control group, Amp B 1x and 2x, and L-Amp1 X and 2X, respectively. A significant reduction in vascular density is seen in both groups that received Amp B and AmBisome. The embryos that received the Amp B exhibited less vascular density than AmBisome (error bars show mean standard error; *p < 0.05 compared control group).

The effect of the Amp B on the chick’s YSM at 24, 48, and 72 h of primary development is presented in Figure 5. There was a significant reduction of vascular density from the vasculature of the treated embryos in both groups given Amp B and AmBisome. Statistical analysis revealed that embryos that received Amp B exhibited less vascular density than the AmBisome (Figure 6).

#### 3.2.2 Gene Expression Results

This study showed that Amp B alone significantly increased the expression of Caspase-8, Apaf1, and Bax and decreased Bcl-2 genes as apoptotic mediators relative to the control group (*p* < 0.05). In the AmBisome group, drugs activate the apoptotic pathway to a lesser extent than the Amp B group drug, so that in all apoptotic genes studied, the expression of genes was lower than in the Amp group.

**FIGURE 7:**
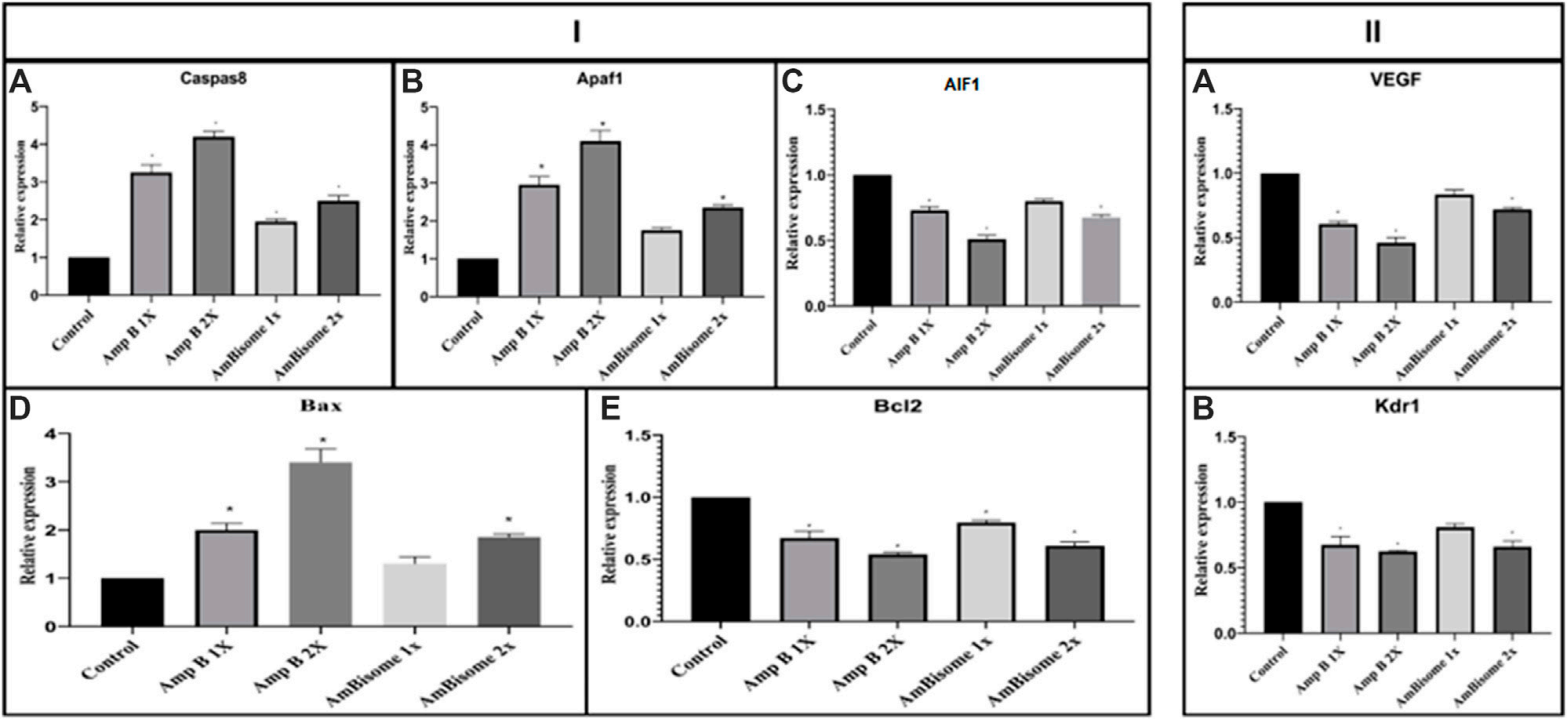
Amp B and AmBisome induced (I) apoptotic mediator and (II) angiogenesis genes in the chick’s extra-embryonic membrane vasculature. (I) The expression level of the apoptotic mediator **(A)** Caspase-8, **(B)** Apaf1, **(C)** AIF1, **(D)** Bax, and **(E)** Bcl2 (II) angiogenesis genes **(A)** VEGF and **(B)** KDR the Amp B -treated embryos compared to controls. The expression levels were normalized to GAPDH and HPRT and calibrated to controls (error bars show standard mean error; *p < 0.05).

Angiogenesis genes (VEGF and KDR) showed, in amp1X and amp2X groups, gene expression lower than the control group (*p* < 0.05) and AmBisome 2X. The effects of this form are less than the usual form of the drug, and the process of angiogenesis is less altered (Figure 7).

#### 3.2.3 IHC Results

**FIGURE 8:**
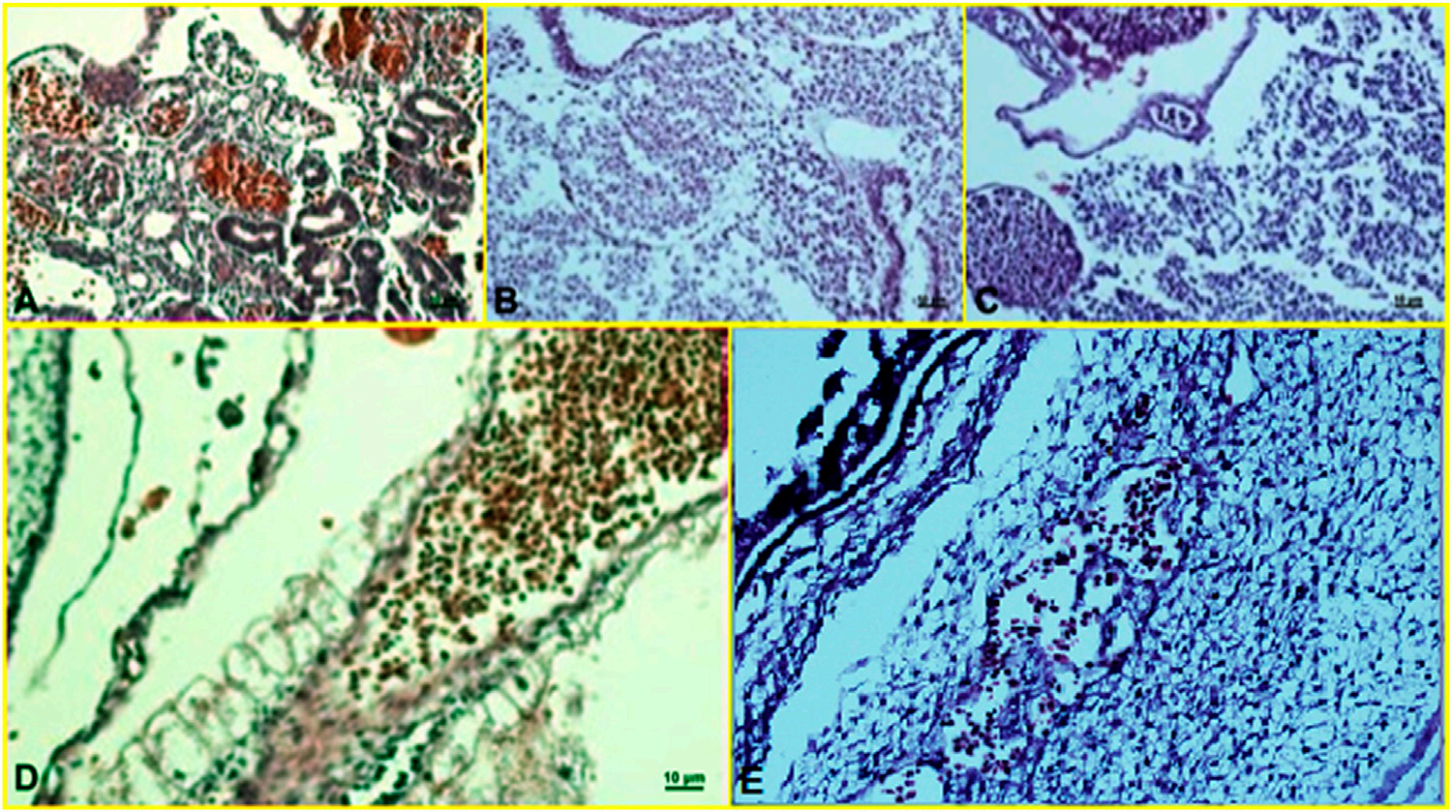
Microscopy images of H&E staining of chick embryo **(A)** control group, **(B)** Amp B 1X,**(C)** Amp B 2X, **(D)** AmBisome 1X, **(E)** AmBisome 2X.

**FIGURE 9:**
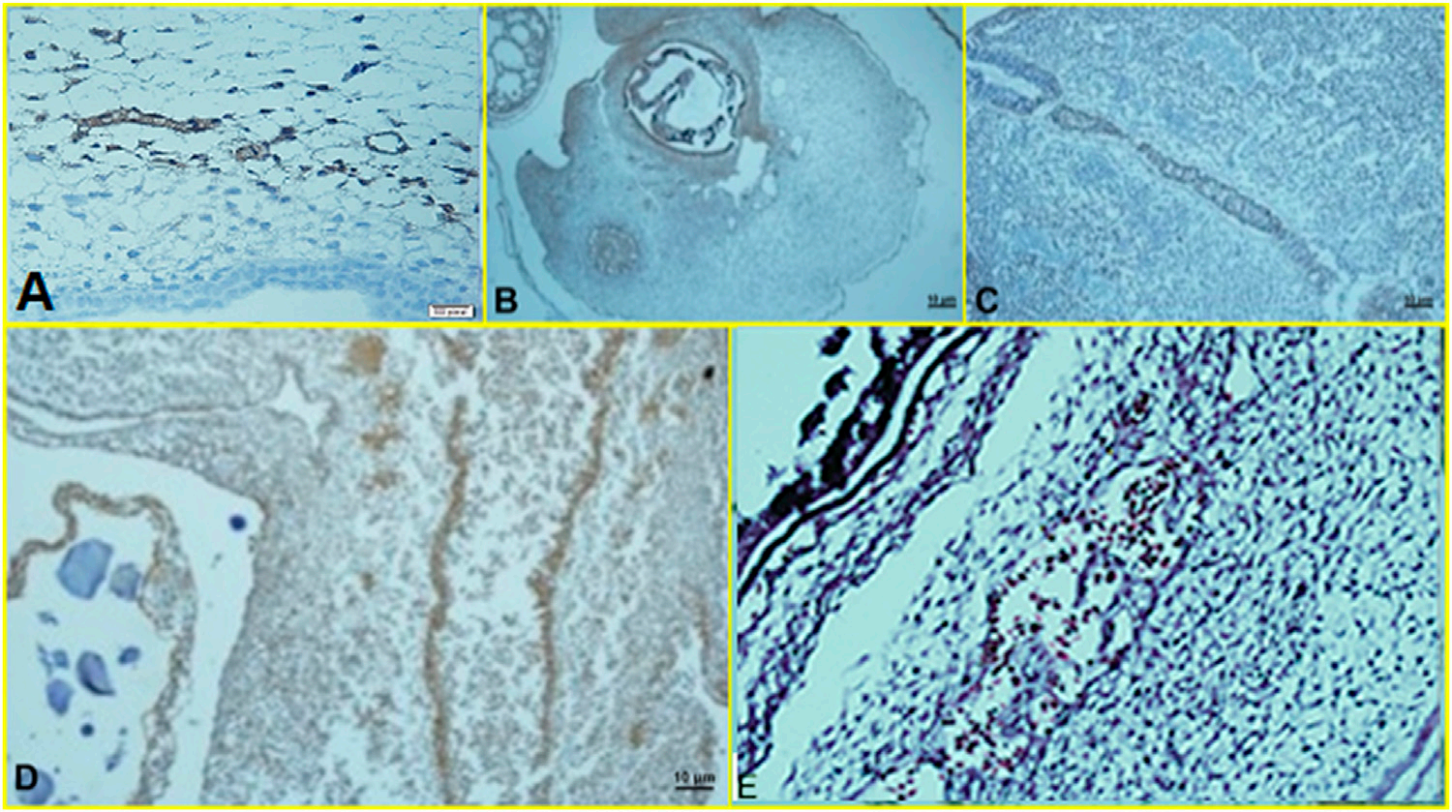
Microscopy images of IHC staining of CD34 from chick embryo **(A)** control group, **(B)** Amp B 1X, **(C)** Amp B 2X, **(D)** AmBisome 1X, **(E)** AmBisome 2X.

**FIGURE 10:**
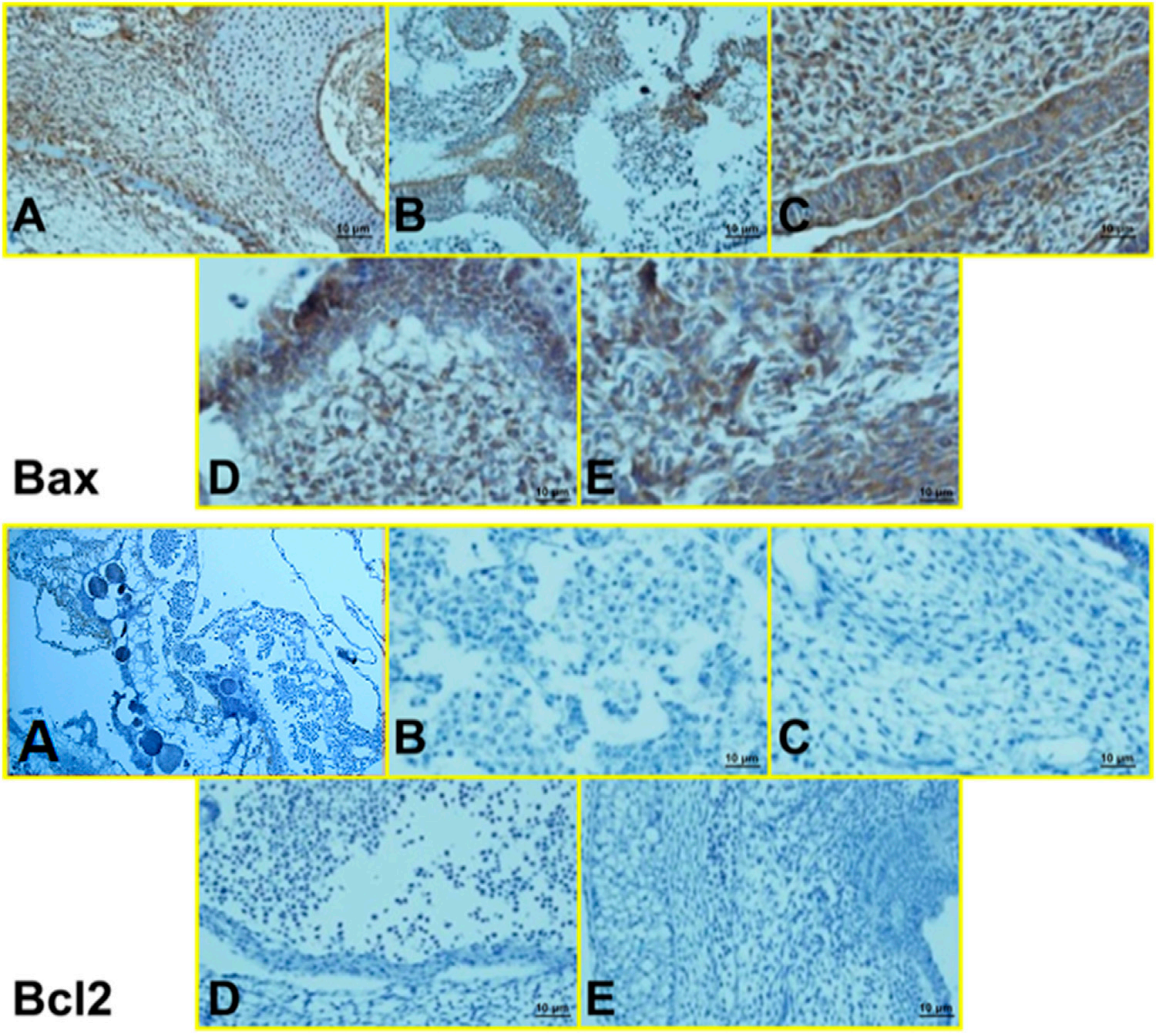
Microscopy images of IHC staining of Bax (up) and Bcl 2 (down) from chick embryo in **(A)** control group, **(B)** Amp B 1X, **(C)** Amp B 2X, **(D)** AmBisome 1X, **(E)** AmBisome 2X (40X).

Histopathological findings, based on comparing different regimens of therapies with control non-treated chicken embryos, showed in the H&E staining a decrease in the collapse of the vascularity and increased degenerative and apoptotic–necrotic changes of the embryonic cells. They were confirmed by immunohistochemical reactions as decreased staining of the vessels by CD34 and increased Bax staining associated with reduced to absent staining for Bcl-2. In order of frequency, the severity of this tissue damage was noted mostly in Amp B 2X, 1X, AmBisome B 2X, and finally AmBisome 1X with minor damage (Figures 8–10).

## 4 Discussion

Leishmaniasis is a public health problem with a worldwide distribution, especially in tropical and sub-tropical areas (Torres-Guerrero et al., 2017). In addition to pentavalent antimonials as a primary treatment against leishmaniasis, Amp B, pentamidine, and miltefosine can be used too (Singh and Sivakumar, 2004). Amp B is a polyene antifungal medication that targets the cell wall of promastigotes and amastigotes by adhering to ergosterol (Stone et al., 2016; Mosimann et al., 2018). The development of lipid formulations leads to reduced Amp B toxicity and increases its uptake by cells of the mononuclear phagocyte system. AmBisome, Amp B lipid compound (Abele), and Amp B colloidal dispersion (Amphocil) are three lipid preparations of Amp B that are available (Yardley and Croft, 1997; Balaña-Fouce, 1998). Both conventional and AmBisome are in group B of the US Food and Drug Administration (FDA) pharmacological drugs.

During pregnancy, some physiological characteristics, such as the immune response change, increase the risk of infectious and parasitic diseases (Wegmann et al., 1993; Mellor and Munn, 2000). Cutaneous leishmaniasis during pregnancy has a different clinical manifestation, such as larger lesions with a highly atypical, exophytic appearance. In addition, an increase in the risk of fetal complications may happen if treatment failure occurs (Morgan et al., 2007). Besides, deferment of VL treatment during pregnancy may lead to severe disease characterized by high-grade anemia (Nyakundi et al., 1988; Pagliano et al., 2005). Therefore, treatment of leishmaniasis as soon as possible during pregnancy is critical. Although the pentavalent antimonial organic compounds have been used for more than 75 years, these compounds have shown antagonistic effects on the normal regulation of apoptotic genes and have deleterious significance based on a previous study (Khosravi et al., 2018).

Drug poisonousness is of critical concern during embryonic development. The exact pathway of embryotoxicity following Amp B and AmBisome application is not clearly understood. We hypothesize that alterations in apoptosis and angiogenesis are two mechanisms involved in the toxicity of Amp B and AmBisome.

Products formulated with lipid afford various potential benefits when compared with native Amp B (Hiemenz and Walsh, 1996): 1) a low soluble drug, related to conservative Amp B deoxycholate, can be provided more efficiently for parenteral infusion if linked to lipids; 2) lipid bilayer encapsulation preserves the drug from obliteration including enzymatic degeneration and host immune factor deactivation; 3) liposomes improve the pharmacokinetic profile of the medication by gently delivering Amp B, resulting in a deviation from potentially susceptible tissues, most prominently, the kidney; 4) the structure of the lipid-mediator assures that Amp B continues related to the carrier, excluding uninhibited drug outflow, and is thus unobtainable to interrelate with mammalian cells to apply its toxic results; and 5) the lipid-carrier promotes the uptake of the complex by the circulating monocytes, as well as other cells of the mononuclear phagocyte system. Therefore, drug delivery can be targeted to desired sites of infection, enhancing the performance of delivery. Selective transfer of the antifungal agent from the donor lipid to the fungal cell membrane may occur (Janknegt et al., 1992; Ng and Denning, 1995).

In this study, the anti-angiogenic properties of Amp B and AmBisome forms were investigated *in silico* and *in vivo* using a chick embryo model. According to studies, the current study evaluates the effectiveness of Amp B and AmBisome in vascular modification by utilizing a chick embryo model. Our admetSAR study determined the evaluation and analysis of all the limits of Amp B and AmBisome. Amp B was unsuitable because of the lower LD50 value separate from other limitations. Prediction of the ADME-Toxicity reveals that AmBisome possesses a better pharmacological effect than Amp B. As a result, AmBisome could demonstrate outstanding base drug contenders as it is a potent, discerning, orally bioavailable, and less poisonous inhibitor. In the present study, we recognized that the best outcome of all the dockings by utilizing the MVD Software was obtained between AmBisome and Bax, Bcl-2, Caspase-8, KDR1, and VEGF-A receptors, followed by Amp B concerning their free binding energy. In contrast, molecular properties of Amp B and AmBisome indicate that owing to less HOMO–LUMO, Egap of AmBisome, the chemical reactivity of AmBisome was higher related to Amp B. The results indicate that AmBisome can be developed as leishmaniasis therapy.

The present *in vivo* findings demonstrated that AmBisome is less toxic and possesses a superior pharmacological action and chemical reactivity than Amp B alone. Hence, this formulation can be an alternative drug used for the treatment of leishmaniasis. These results are consistent with the previous report that suggested liposomal amphotericin B was the preferred therapy choice for the management of VL in pregnant women (Mishra et al., 2007; Solomon et al., 2007; Dahal et al., 2021).

This article looked at apoptotic-control genes such as Caspase family proteins (CASP), apoptotic protease activating factor 1 (Apaf1), and apoptosis-inducing factor 1 (AIF1), as well as Bax and Bcl-2 genes. Bcl-2 can inhibit cell death. In comparison, Bax overexpression can speed up cell death (Gil-Gómez et al., 1998). On the contrary, changes in the expression of the VEGF gene can be effective in the progression of the diseases.

According to the findings, the relative expression levels of some apoptotic-regulating genes, including Caspase-8 and Apaf1, increased dramatically in the drug-exposed extra-embryonic membrane (EEM) in a dose-dependent manner. AIF1, as a pro-apoptotic family member, acts as an independent Caspase. It might be released from the mitochondria (Strzyz, 2017). In confirming the gene expression of apoptosis-related markers, the IHC results may indicate an increase in the occurrence of apoptosis in the cell. In addition, changes in the CD34 staining confirm changes in the pattern of angiogenesis.

In the present investigation, our vascular analysis revealed that vascular density following Amp B and AmBisome was reduced in both groups, but the embryos that received the Amp B exhibited less vascular density than the AmBisome. Amphotericin B is a unique drug as it is present in different preparations to be used as a clinically important agent against infectious diseases, including protozoal and fungal pathogens (Jafari et al., 2021; Ubals et al., 2021). In the last decades, conventional Amp B was the single antifungal agent available for the treatment of invasive fungal infections. However, significant dose-limiting adverse reactions caused the stimulus to develop liposomal amphotericin B, which has been used for 25 years to treat a wide range of fungal and leishmanial infections (Cavassin et al., 2021).

Based on these results, it can be suggested that using an AmBisome compared to the non-liposomal form in pregnant women has less toxic effects on the fetus. As far as we are aware, this is a unique study to target the different aspects of Amp B and AmBisome toxicity with a chick embryo model.

Our results indicate that Amp B exerts its cytotoxicity effect on the chick embryo. This drug should be considered embryotoxic during pregnancy, while in AmBisome, it is released slowly and remains safe. As a result, the use of Amp B complexes during pregnancy should be regarded as potentially embryotoxic, at least until more data on safety for the human fetus is available. Therefore, medication use should be restricted during pregnancy or only agreed to when the benefit outweighs the risk, particularly in rural areas. However, further evidence-based studies are crucial to precisely indicate the teratogenic effect of Amp B and AmBisome implications for the embryo during pregnancy using a suitable animal model in future experimental settings.

## Data Availability

The original contributions presented in the study are included in the article/Supplementary Materials. Further inquiries can be directed to the corresponding author.
