# Supplementary figures and images for: Cytotoxicity of Amphotericin B and AmBisome: In Silico and In Vivo Evaluation Employing the Chick Embryo Model
Source: Front Pharmacol. 2022 Jun 8;13:860598. doi: 10.3389/fphar.2022.860598 (PMC9214246; doi:10.3389/fphar.2022.860598)

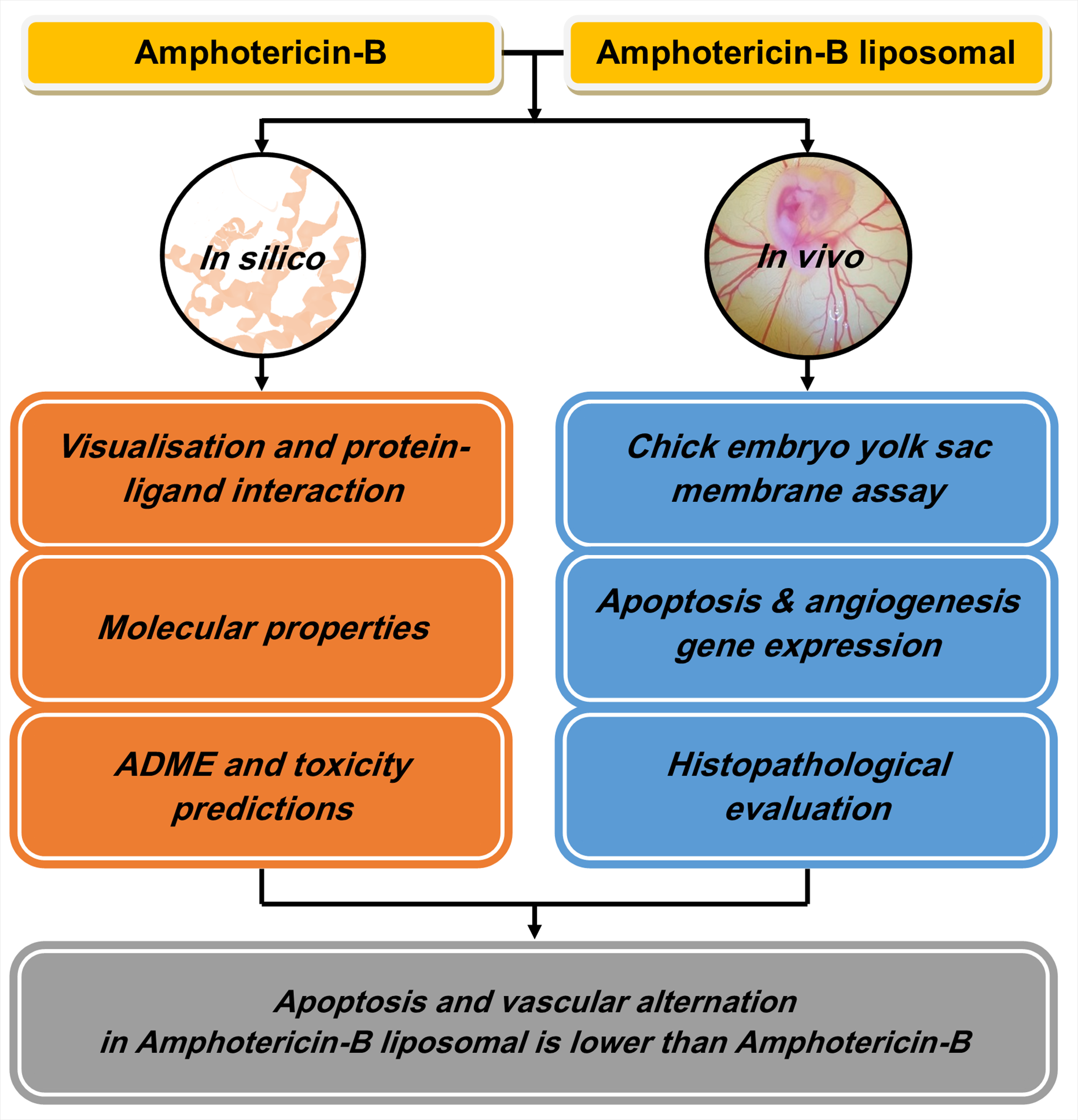

Supplement: Supplementary file 1 [file Image1.TIF]
